# Frequency of Quarterly Self-reported Health-Related Social Needs Among Older Adults, 2020

**DOI:** 10.1001/jamanetworkopen.2022.19645

**Published:** 2022-06-30

**Authors:** Nancy Haff, Niteesh K. Choudhry, Gauri Bhatkhande, Yong Li, Dana Drzayich Antol, Andrew Renda, Julie C. Lauffenburger

**Affiliations:** 1 Center for Healthcare Delivery Sciences, Division of Pharmacoepidemiology and Pharmacoeconomics, Department of Medicine, Brigham and Women’s Hospital and Harvard Medical School, Boston, Massachusetts; 2 Healthcare Research, Humana, Louisville, Kentucky; 3 Bold Goal & Population Health Strategy, Humana, Louisville, Kentucky

## Abstract

This cohort study analyzes survey responses regarding social needs throughout 2020 among Medicare Advantage plan beneficiaries.

## Introduction

Approximately half of US adults report a health-related social need (HRSN), such as food and housing,^1,2^ but less is known about how HRSNs unfold over time. The goal of this cohort study was to describe quarterly changes in HRSNs among a Medicare Advantage cohort.

## Methods

This cohort study was approved by the Mass General Brigham Institutional Review Board, which waived the informed consent requirement because of the secondary use of deidentified data. We followed the STROBE reporting guideline.

We used data from a longitudinal nationwide survey of individuals with continuous enrollment in Humana Medicare Advantage plans.^3^ Research-eligible beneficiaries 65 years or older who completed an initial HRSN survey in quarter 1 of 2020 were sent follow-up surveys for the remaining quarters of 2020. Questions assessed 8 HRSNs: financial strain, food insecurity, loneliness, caregiving needs, housing insecurity, poor housing quality, utility insecurity, and unreliable transportation (eMethods in the Supplement). Analyses included only participants who responded to all 4 surveys (eTable in the Supplement). The HRSN ratings were reduced to binary variables for each need.

Frequency of each HRSN and proportion with 1 or more HRSN were calculated in each quarter. A Sankey plot was used to visualize participants transitioning among total numbers of HRSNs between quarters. We used enrollment data and *International Statistical Classification of Diseases and Related Health Problems, Tenth Revision,* diagnosis codes to describe baseline characteristics of participants in 3 distinct groups: (1) no HRSN across all quarters, (2) any fluctuation between 0 and 1 or more HRSNs, and (3) 1 or more HRSNs across all quarters. Race and ethnicity were identified from insurance enrollment records. Statistical analyses were performed in RStudio, version 1.3.1073 (RStudio).

## Results

In total, 16 102 of 68 133 eligible beneficiaries (23.6%) responded to surveys in all 4 quarters and were included in this analysis. Participants had a mean (SD) age of 74 (5.1) years; 9558 (59.4%) were women, and 6544 (40.6%) were men (Table).

**Table.** Participant Characteristics by Changes in Health-Related Social Needs Across 4 Survey Quarters

Characteristic	Participants, No. (%)			
	No need (n = 4979)	Changing needs (n = 7634)	Consistent ≥1 need (n = 3489)	Total (n = 16 102)
Age, mean (SD), y	74.22 (5.52)	74.55 (5.88)	72.84 (5.65)	74.08 (5.76)
Race and ethnicity^a^				
Black	425 (8.5)	1306 (17.1)	923 (26.5)	2654 (16.5)
White	4361 (87.6)	6056 (79.3)	2433 (69.7)	12 850 (79.8)
Other^b^	99 (2.0)	187 (2.5)	93 (2.7)	379 (2.4)
Unknown	94 (1.9)	85 (1.1)	40 (1.2)	219 (1.4)
Sex				
Female	2667 (53.6)	4559 (59.7)	2332 (66.8)	9558 (59.4)
Male	2312 (46.4)	3075 (40.3)	1157 (33.2)	6544 (40.6)
Geographic region				
Northeast	140 (2.8)	278 (3.6)	119 (3.4)	537 (3.3)
Midwest	1291 (25.9)	1936 (25.4)	740 (21.2)	3967 (24.6)
South	2976 (59.8)	4655 (61.0)	2349 (67.3)	9980 (62.0)
West	572 (11.5)	765 (10.0)	281 (8.1)	1618 (10.0)
Population density				
Urban	3206 (64.4)	4774 (62.5)	2253 (64.6)	10 233 (63.6)
Suburban	1226 (24.6)	1904 (24.9)	788 (22.6)	3918 (24.3)
Rural	439 (8.8)	807 (10.6)	389 (11.2)	1635 (10.2)
Unknown	108 (2.2)	149 (2.0)	59 (1.7)	316 (2.0)
SVI, mean (SD)^c^	0.44 (0.26)	0.51 (0.27)	0.58 (0.26)	0.50 (0.27)
Gagne comorbidity score, mean (SD)^d^	1.48 (2.36)	2.20 (2.73)	2.77 (2.89)	2.10 (2.70)
No. of days hospitalized, mean (SD), d	0.56 (2.77)	0.87 (3.65)	1.03 (3.84)	0.81 (3.45)
No. of ED visits, mean (SD)	0.39 (1.16)	0.61 (1.50)	0.78 (1.73)	0.58 (1.47)
No. of office visits, mean (SD)	11.33 (10.29)	12.72 (11.66)	13.46 (11.88)	12.45 (11.33)
No. of unique medications filled, mean (SD)	7.56 (5.30)	9.38 (6.29)	11.39 (7.04)	9.25 (6.33)
Metastatic cancer	50 (1.0)	109 (1.4)	62 (1.8)	221 (1.4)
CHF	607 (12.2)	1406 (18.4)	837 (24.0)	2850 (17.7)
Dementia	71 (1.4)	205 (2.7)	77 (2.2)	353 (2.2)
Kidney failure	961 (19.3)	1836 (24.1)	930 (26.7)	3727 (23.2)
Weight loss	126 (2.5)	282 (3.7)	157 (4.5)	565 (3.5)
Hemiplegia	31 (0.6)	69 (0.9)	44 (1.3)	144 (0.9)
Alcohol disorder	131 (2.6)	202 (2.7)	104 (3.0)	437 (2.7)
Any tumor	608 (12.2)	1052 (13.8)	440 (12.6)	2100 (13.0)
Cardiac arrythmias	1199 (24.1)	2031 (26.6)	955 (27.4)	4185 (26.0)
Chronic pulmonary disease	962 (19.3)	2207 (28.9)	1326 (38.0)	4495 (27.9)
Coagulopathy	197 (4.0)	362 (4.7)	188 (5.4)	747 (4.6)
Complicated diabetes	850 (17.1)	1979 (25.9)	1199 (34.4)	4028 (25.0)
Deficiency anemia	679 (13.6)	1340 (17.6)	760 (21.8)	2779 (17.3)
Fluid and electrolytes	450 (9.0)	924 (12.1)	484 (13.9)	1858 (11.5)
Liver disease	260 (5.2)	402 (5.3)	252 (7.2)	914 (5.7)
Peripheral vascular disease	1065 (21.4)	2279 (29.9)	1171 (33.6)	4515 (28.0)
Psychiatric condition	578 (11.6)	1565 (20.5)	1086 (31.1)	3229 (20.1)
Pulmonary disorders	165 (3.3)	424 (5.6)	242 (6.9)	831 (5.2)
HIV or AIDS	4 (0.1)	15 (0.2)	11 (0.3)	30 (0.2)
Hypertension	3601 (72.3)	6049 (79.2)	2873 (82.3)	12 523 (77.8)

Abbreviations: CHF, congestive heart failure; ED, emergency department; SVI, social vulnerability index.

^a^ Race and ethnicity data were identified from insurance enrollment records.

^b^ Other category included American Indian or Alaska Native, Asian, Asian American or Pacific Islander, and Hispanic individuals.

^c^ SVI score range: 0 to 1, with higher scores indicating greater social vulnerability.

^d^ Gagne comorbidity score range: −2 to 20, with higher scores indicating greater illness severity.

At the population level, the prevalence of each HRSN and reporting of 1 or more HRSNs (lowest-highest prevalence: 42.9%-45.2%) were mostly consistent over time. Financial strain (30.4%-32.8%), poor housing quality (17.2%-18.8%), and food insecurity (17.1%-18.5%) were the most frequent needs across all quarters.

At the individual level, participants reported substantial fluctuations (Figure): 30.9% (n = 4979) had no HRSN across all quarters, 47.4% (n = 7634) had between 0 and 1 or more HRSNs, and 21.7% (n = 3489) had 1 or more HRSNs at all time points. Female sex, Black race, residency in the South, and higher comorbidity burden were disproportionately represented in those reporting 1 or more HRSNs, followed by those with fluctuating needs (Table).

**Figure.**
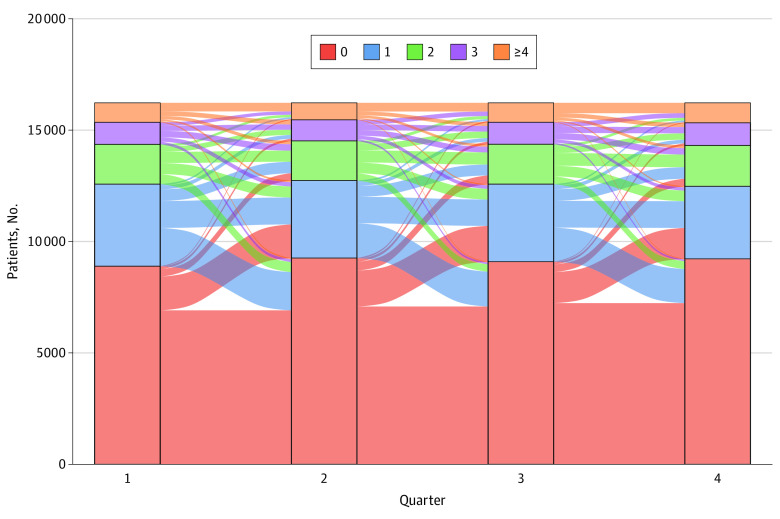
Fluctuations in Number of Health-Related Social Needs Reported by Individual Patients Across Quarters

## Discussion

In this sample of Medicare Advantage plan beneficiaries, prevalence of HRSNs was consistent over time and similar to previous assessments.^4,5^ However, at the individual level, we observed marked fluctuations in HRSNs across quarters. Previous research identifying the average needs of a population at 1 time point may miss the dynamic nature of individual-level HRSNs observed in this study.

The survey response rates, Medicare Advantage sample, and measurement during the COVID-19 pandemic (when both HRSNs and services to mitigate them change rapidly) may have limited the generalizability of the findings. Specifically, restricting analyses to those who responded in all quarters could have underestimated social needs, although the characteristics between such participants and nonrespondents were similar.

Nonetheless, the findings could have important implications for health systems and communities that wish to offer interventions to address HRSNs. First, in some populations, more frequent HRSN screening may need to be conducted. Second, support, such as connection to community resources, may need to be offered immediately as needs change. Third, key demographic and clinical characteristics appear to differ between individuals with consistent vs fluctuating HRSNs, and these differences could help identify those who could benefit the most from targeted interventions.

## Notes

Supplement.
eMethods
**eTable.** Characteristics of Patients in Full Sample, Those Who Responded to 2-3 Survey Timepoints, and Those Who Responded to All Four Study Timepoints
eReferences
