# Supplementary material for: Frequency of Quarterly Self-reported Health-Related Social Needs Among Older Adults, 2020
Source: JAMA Netw Open. 2022 Jun 30;5(6):e2219645. doi: 10.1001/jamanetworkopen.2022.19645 (PMC9247734; doi:10.1001/jamanetworkopen.2022.19645)
Supplement: Supplement. — eMethods eTable. Characteristics of Patients in Full Sample, Those Who Responded to 2-3 Survey Timepoints, and Those Who Responded to All Four Study Timepoints eReferences [file jamanetwopen-e2219645-s001.pdf]

# Supplementary Online Content

Haff N, Choudhry NK, Bhatkhande G, et al. Frequency of quarterly self-reported health-related social needs among older adults, 2020. *JAMA Netw Open*. 2022;5(6):e2219645. doi:10.1001/jamanetworkopen.2022.19645

**eMethods.**

**eTable.** Characteristics of Patients in Full Sample, Those Who Responded to 2-3 Survey Timepoints, and Those Who Responded to All Four Study Timepoints

**eReferences**

This supplementary material has been provided by the authors to give readers additional information about their work.

© 2022 Haff N et al. *JAMA Network Open.*

## eMethods

Surveys were developed using a subset of questions from the widely-validated CMS Accountable Health Communities HRSN Screening tool.[1] Non-institutionalized adults enrolled in an individual Medicare Advantage plan offered by Humana Inc. as of October 1st 2019 were invited to complete the survey by interactive voice response phone calls, text messages, or email, depending on the availability of contact information. If multiple individuals were eligible within the same household, one individual was randomly selected for participation. Individuals were asked about financial strain, food insecurity, loneliness or social isolation, caregiving needs, housing insecurity, poor housing quality, utility insecurity, and unreliable transportation. The survey was offered in English and Spanish. The initial survey was sent to 431,215 individuals between November 2019 and February 2020 for 2020Q1 and the results have been previously published.[2] Of this sample, 68,133 responded, were age 65 and older, remained continuously enrolled in their Medicare Advantage plan, were not contractually excluded from research, and were sent subsequent surveys. These follow-up surveys were sent April-June 2020 for Q2, August-October 2020 for Q3 and October 2020-January 2021 for Q4.

We also compared the observed demographic and clinical characteristics among those who responded to a) only the first survey, b) to 1-2 additional survey timepoints, and c) to our cohort who completed all survey timepoints to determine the potential impact of response rate on generalizability (**eTable**). We found that baseline demographic characteristics were very similar across the groups with standardized mean differences all <0.1, indicating that this group of respondents is strongly generalizable to the overall sample.

For this study, survey responses were reduced to binary variables for each need. In the case where questions had multiple response options, any response indicating any degree of need

© 2022 Haff N et al. *JAMA Network Open.*

was counted as that need being present, with the exception of loneliness or social isolation, where answers to the question “How often do you feel lonely of isolated from those around you?” responses of “never,” “rarely,” and “sometimes” were counted as no need, and “often” and “always” were counted as a need, consistent with prior work. Patient demographic data was extracted from the Medicare Advantage enrollment files, and International Classification of Disease (ICD) 9 and 10 codes were used to assess for comorbid conditions, health system utilization, and calculate the Gagne comorbidity score.[3]

We used the *‘ggalluvial’* package, an extension of the *‘ggplot2’* package in R, to create the Sankey plot.[8] The colored vertical nodes represent the number of health-related social needs. The height of the bands connecting the nodes represents the volume of patients transitioning between each node. The change in the number of health-related social needs is represented by the splitting and merging of the bands between two quarters.

© 2022 Haff N et al. *JAMA Network Open.*

**eTable.** Characteristics of Patients in Full Sample, Those Who Responded to 2-3 Survey Timepoints, and Those Who Responded to All Four Study Timepoints

| Patient Characteristics (N, %) | Full Sample | Responded in 2 or 3 quarters | Responded in all 4 quarters (N, %) |
|---|---|---|---|
| | (N = 68,133) | (N = 32,163) | (N = 16,102) |
| Age (mean, sd) | 74.03 (±6.62) | 74.41 (±5.82) | 74.08 (5.76) |
| Female | 37026 (54.4%) | 18683 (58.1%) | 9558 (59.4) |
| Race | | | |
| White | 52492 (77.1%) | 25458 (79.2%) | 12850 (79.8) |
| Black | 10955 (16.1%) | 5228 (16.3%) | 2654 (16.5) |
| Other | 3696 (5.4%) | 1082 (3.4%) | 379 (2.4) |
| Unknown | 963 (1.4%) | 395 (1.2%) | 219 (1.4) |
| Geographic Region | | | |
| Northeast | 2046 (3.0%) | 1017 (3.2%) | 537 (3.3) |
| Midwest | 14907 (21.9%) | 7514 (23.4%) | 3967 (24.6) |
| South | 42158 (61.9%) | 20559 (63.9%) | 9980 (62.0) |
| West | 8995 (13.2%) | 3073 (9.6%) | 1618 (10.0) |
| Population Density | | | |
| Urban | 42550 (62.5%) | 19535 (60.7%) | 10233 (63.6) |
| Suburban | 16746 (24.6%) | 8301 (25.8%) | 3918 (24.3) |
| Rural | 7350 (10.8%) | 3689 (11.5%) | 1635 (10.2) |
| Unknown | 1459 (2.1%) | 638 (2.0%) | 316 (2.0) |

**Note**: Continuous eligibility was required from the baseline assessment period for the full sample, but additional eligibility was required through 2020 for the groups that responded to more than one timepoint, which resulted in a smaller number of individuals across those two groups when compared to the full sample. The demographic characteristics of the full sample are included in the table to assist with interpretation of any potential influence from this additional attrition.

© 2022 Haff N et al. *JAMA Network Open.*

**eReferences**

1. Billioux A, Verlander K, Anthony S, Alley D. Standardized Screening for Health-Related Social Needs in Clinical Settings: The Accountable Health Communities Screening Tool. *NAM Perspect*. Published online May 30, 2017. doi:10.31478/201705b

2. Long CL, Franklin SM, Hagan AS, et al. Health-Related Social Needs Among Older Adults Enrolled In Medicare Advantage. *Health Aff (Millwood)*. 2022;41(4):557-562. doi:10.1377/hlthaff.2021.01547

3. Gagne JJ, Glynn RJ, Avorn J, Levin R, Schneeweiss S. A combined comorbidity score predicted mortality in elderly patients better than existing scores. *J Clin Epidemiol*. 2011;64(7):749-759. doi:10.1016/j.jclinepi.2010.10.004

© 2022 Haff N et al. *JAMA Network Open.*
